# Acceptability and understanding of the Ages & Stages Questionnaires®, Third Edition, as part of the Healthy Child Programme 2‐year health and development review in England: Parent and professional perspectives

**DOI:** 10.1111/cch.12639

**Published:** 2019-02-20

**Authors:** Sally Kendall, Avril Nash, Andreas Braun, Gonca Bastug, Emeline Rougeaux, Helen Bedford

**Affiliations:** ^1^ Centre for Health Services Studies University of Kent Canterbury UK; ^2^ Centre for Research in Primary and Community Care University of Hertfordshire Hatfield UK; ^3^ Children's Policy Research Unit, Institute of Child Health University College London London UK

**Keywords:** ASQ‐3, child development 2 years, health professionals, Healthy Child Programme, parent perceptions

## Abstract

**Background:**

The Healthy Child Programme is the universal public health system in England to assess and monitor child health from 0 to 19. Following a review of measures for closer monitoring at age 2 years, the Department of Health for England implemented the Ages & Stages Questionnaires®, Third Edition (ASQ‐3™; Hereon, ASQ‐3).

**Aim:**

The aim of this study was to evaluate the acceptability and understanding of the ASQ‐3 in England by health professionals and parents.

**Method:**

A mixed‐methods approach was used. This paper reports on the qualitative data drawn from interviews with 40 parents and 12 focus groups with 85 health professionals. The data were analysed using applied thematic analysis.

**Findings:**

Overall, parents and health professionals found the ASQ‐3 acceptable and understandable and could use it as a measure at age 2 years. The ability to work in partnership was valued.

Some limitations included potential to cause anxiety, concerns around the safety of some of the items, and use of Americanized language. Health professional's training in the use the ASQ‐3 was inconsistent.

**Conclusion:**

The ASQ‐3 is an acceptable and understandable measure to use as part of the 2‐year assessment with some adaptations to the English context and some standardized training for health professionals.

**Key messages**

- The ASQ‐3 is acceptable and understandable as measure of child development at age 2–2.5 years by parents and health professionals in England.
- Parents and health professionals appreciate the partnership approach that the ASQ‐3 offers.
- For some parents, the ASQ‐3 is seen as “test,” and it can raise anxiety, which may lead to distortion in completion of the measure.
- There is variation across England in the way in which the ASQ‐3 is used by health professionals that needs to be addressed in order for the ASQ‐3 to be a true population measure.
- There is a need for further training in using and scoring the ASQ‐3 by health professionals.

## 1 INTRODUCTION AND BACKGROUND

In England, the Healthy Child Programme (HCP) is the universal public health programme offered to all children and families. It comprises health and development reviews, health promotion, parenting support, screening, and immunization programmes with a review of health and development at 2 years of age (Department of Health, 2009). In 2011, in response to a series of expert reports that highlighted the importance of the early years in shaping a child's future life chances (Allen, 2011; Field, 2010), the Government at that time stated their intention to develop an outcome measure of child development linked to the HCP at 2 to 2.5 years of age (Department of Education and Department of Health, 2011).

A review (Bedford, Walton, & Ahn, 2013) of existing measures was commissioned to identify the most suitable for this purpose. A total of 32 measures were reviewed (see Bedford et al., 2013, for all references), and the Ages & Stages Questionnaires®, Third Edition (ASQ‐3™; Squires et al., 2009), was identified as the existing measure most able to provide data for the public health outcome measure and suitable for inclusion in the 2‐year review. Although ASQ‐3 was being used as part of the Family Nurse Partnership programme in some areas of England, little was known about its acceptability by health professionals (HPs) and parents in the United Kingdom. Other studies have investigated the feasibility and acceptability of the use of the ASQ measure in other countries; for example, in Chile, parents found the ASQ acceptable, although this was not an in‐depth qualitative account (Armijo, Schonhaut, & Cordero, 2015). Before the ASQ‐3 was introduced across England, it was considered important to explore the extent to which parents and HPs would find the measure understandable and acceptable. Because the ASQ‐3 is a parent‐completed measure, a higher degree of uptake would be expected if parents find it both acceptable and understandable. From a public health perspective, the uptake of a measure of development is important in relation to the monitoring and identification of trends in child development and inequalities across different types of communities.

The ASQ‐3 was originally developed by Squires and colleagues in the United States as a part of a suite of measures for screening for developmental delay across the age range from 2 months to 5 years. The 24‐, 27‐, and 30‐month versions were considered to be suitable for 2‐ to 2.5‐year‐old children in England. The ASQ‐3 questionnaires are theoretically developed from the Bayley scales of child development (Bayley, 2005). The ASQ‐3 assesses fine and gross motor skills, communication, problem solving, and personal‐social aspects of development on the basis of parents' own observations of their child. Studies in the United States and other parts of the world have shown them to be reliable and valid as a screening tool, although the specificity and sensitivity are largely based on U.S. norms (Janson & Squires, 2004; Singh, Jung Yeh, & Blanchard, 2017). For this reason, the Department of Health for England was concerned that the ASQ‐3 would be an *indicator* of development at 2 years, not a screening test. As the 2‐year review was being developed by the Department of Health for England, the Department of Education was also reviewing children's development as they approached nursery education under their Early Years programme; this resulted in a separate child assessment taking place in Early Years settings, and a decision was made to integrate the two assessments to provide both health and education data. The so‐called integrated review was being carried out in some local authorities at the time this study was conducted.

### 1.1 Aims and objectives

Given the public health requirement in England to introduce a population measure of child development at 2 years, the overarching aim of this study was to determine how acceptable and understandable the ASQ‐3™: A Parent‐Completed Child Monitoring System (Squires et al., 2009) is to parents and professionals for assessing the development of 2‐year‐olds. The objectives were twofold: to determine acceptability and understanding among (a) parents whose children had had a HCP 2‐year review and (b) HPs who were using the measure as part of the HCP 2‐year review.

## 2 METHODS

Taking a mixed‐methods approach, this exploratory study used interviews, focus groups, and survey methods to address the aims and objectives. In this paper, we report on the qualitative component of the study as this provides an in‐depth view of how parents and HPs understand, use, and accept the ASQ‐3 measure. Further details are in the full report (Kendall et al., 2014).

### 2.1 Public engagement with the study

Because this study was directly concerned with the perspectives of parents, it was integral to the study that we engaged with parents in the development of the study. Patient and public involvement has been documented as central to undertaking research that concerns users of the health services (Wilson et al., 2018). During the design phase of the study, 10 volunteers from the National Children's Bureau parents' group took part in discussions on the proposed research during a half‐day event and put forward suggestions and comments that were implemented wherever practicable. As a result, the final protocol was informed by parent experience and knowledge.

### 2.2 Ethics

Applications were made to research ethics committees at both the University of Hertfordshire and University College London, and to the National Research Ethics Service. All parties advised that the study would be regarded as a service evaluation and, as such, did not require ethical approval but would require normal governance approvals with each National Health Service (NHS) Trust involved and that approval was sought and obtained from the four health trusts.

### 2.3 Study sites

Four study sites were purposively selected and approached using information gathered from the child and maternal health public health observatory data on child health profiles. Inclusion criteria were as follows:

- known to have implemented the HCP 2‐year review;
- known to be using ASQ‐3 as part of the 2‐year review;
- included a variety of geographical locations (North vs. South England);
- included inner London and rural and more mixed locations;
- included an ethnically diverse population; and
- included a range of socio‐economic backgrounds.

Exclusion criteria were as follows: The five local authorities and five pilot partner sites in which implementation of the integrated review was being tested were excluded to avoid overburdening them, but also, by including different sites, we hoped to gain a wider view of practice.

Health Visiting Services at the NHS Community Trusts in the four study sites were contacted and recruited to the study, providing access to both HPs and parent participants. All four sites agreed to take part.

### 2.4 Sample

Forty mothers took part in one‐to‐one interviews. Whilst both mothers and fathers were invited, all interviewees were mothers. The interviews were undertaken by all members of the research team, each taking responsibility for a geographical area. Twelve focus groups were conducted with a total of 85 HPs comprising mainly health visitors and community nursery nurses; one administrator and one staff nurse were also included. The focus groups were facilitated and conducted by members of the research team. Based on Creswell and Creswell's (2017) approach to qualitative sampling, it was considered that 40 in‐depth interviews and 12 focus groups would be adequate to achieve data saturation and data convergence.

## 3 MATERIALS

A semistructured interview was developed for the one‐to‐one parent interviews. This explored parents' expectations of the review, their opinion of the ASQ‐3, ASQ‐3 specific items, and how they evaluated the ASQ‐3 in terms of acceptability, usefulness, ease of use, and their understanding. Questions covered the specific domains of the ASQ‐3 and more general perspectives; for example, “How do you feel this questionnaire works for parents and children?”

Similarly, a semistructured interview schedule was created for the HPs' focus groups. This elicited information about the process for using the ASQ‐3 and 2‐year review and gathered views about its acceptability and usefulness; for example, “How confident are you that this measure gives a comprehensive picture of the 2 year old?”

### 3.1 Procedure

With the support of health visitor managers and administrators in the NHS Community Trusts, parents whose children had recently had their 2‐year review were identified and contacted by post with a questionnaire about their experiences of using the ASQ‐3. The questionnaire included at its end an invitation to take part in a one‐to‐one interview. Forty parents (10 from each area) were purposively selected for interview from 88 parents who were willing to be interviewed. The 88 were responders to the survey element of this study (see Kendall et al., 2014), from a total 153 respondents. The selection reflected, as far as possible, a range of socio‐demographic characteristics, ethnic backgrounds, and education. Parents were consented at interview and received a £20 supermarket voucher in recognition of their interest and the time and effort required to take part. Interviews of around 30–40 min took place largely in parents' homes (*n* = 37), with a further three conducted by telephone.

Focus groups with HPs were arranged via the NHS Trust managers, and consent was received from all HPs taking part. The HPs were not offered an incentive as this was considered to be part of their professional role conducted in work time. Focus groups and all interviews, except one, were audio‐recorded and professionally transcribed. One parent did not wish to be recorded, and written notes were made instead.

### 3.2 Analysis

All data were subjected to applied thematic analysis (Guest, MacQueen, & Namey, 2012), which “focuses on identifying and describing both implicit and explicit ideas within the data” (p. 10). The themes were identified initially through independent open coding of the transcripts undertaken by A. B. and G. B. The research team as a whole discussed the initial codes and coding structure. Disagreements were resolved by A. N. and S. K. through discussion and going back to the data.

The initial categories employed in the analysis were derived from the research question, and the coding structure was developed to address themes of acceptability, utility, and understanding of the ASQ‐3. Further themes were added to take account of emerging topics raised by the research participants to maximize our understanding of their perspective, for example, the theme of challenges raised by the ASQ‐3.

Interview data were coded using MAXQDA 11 qualitative data analysis software, which assessed intercoder agreement at 82%.

Four of the researchers are parents, at different stages in their life courses, who may have had some bearing on their coding. However, as experienced qualitative researchers, this was not considered a significant threat to validity.

### 3.3 Findings

This paper reports on specific findings related to acceptability and understanding of the ASQ‐3 of interest to child HPs. For the full account of the findings, please refer to Kendall et al. (2014).

### 3.4 Acceptability

In general, most parents and HPs accepted the ASQ‐3 as a measure that provides useful information about a child's development at 2 years. Parents enjoyed and found it valuable to observe their own child prior to an assessment visit either in a clinic or at home.

I'm not really sure where she should be at this stage, so it was useful tool to go through … . quite reassuring too. (Parent)

It was fun … so we had our afternoon together … and I enjoyed that, being able to let her participate in doing it, it felt like a fun thing to do instead of another form I have to fill in. (Parent)

… actually amazed at all the things he could do. (Parent)

Parents and HPs were equally positive about the opportunity to work in partnership in relation to the child's development.

With this you are working together with parents … you are encouraging the parents to have their own assessment with their child and see where they are before they come and see you. (HP)

### 3.5 Challenges of ASQ‐3

Parents and professionals did, however, raise some concerns about specific questions in the ASQ‐3. For example, some were deemed inappropriate due to potential safety considerations (e.g., climbing on a chair; flicking switches; threading beads); some appeared to lack a rationale (e.g., “say seven three”; line up four blocks in a row), and there were some repetitive questions, which might affect a child's score (kicking a ball; climbing stairs). There was also some confusion around the meaning of the “sometimes” response to the questions.

The origin of the ASQ‐3 drew comments from several parents and professionals (“oh, another American thing!”), as did some of the American English terms: Fine, apart from the fact it was asking some American things.

One mother echoed others in her the wish that it “was tailored at least to children in this country.”

### 3.6 Potential for anxiety

Despite parents generally reporting that they liked the questionnaire, the findings suggest its use may create anxiety unnecessarily in parents. Some parents indicated that they had been worried before or during completion of the ASQ‐3, using terms such as “nervous,” “over the top,” “paranoid,” and “anxious” in their interviews.

I did worry, cos I thought … when I read through the questions, I thought he had to do it, all of them, and I thought, “Oh, my God, he's really slow.” (Parent)

Parents perceived the ASQ‐3 as a “test” and worried that their child might “fail”; regularly ticking “not yet” caused most anxiety. Moreover, even though parents knew their child well, the specific and detailed questions forced them to reassess and some found this discomforting, as one mother elaborated:

I thought it might put some people off, … you're trying to look at what your child can do … rather than somebody asking “oh can they do this,” it's sort of like there in black and white and if your child can't do that maybe you feel a bit, you know, like there's something wrong with them. (Parent)

HPs, too, had similar concerns, particularly when a child is close to reaching a particular milestone. They talked of parents feeling disempowered if their child is unable to do something when, in reality, as professionals, they would regard the abilities as within the normal range of development:

I've had some parents who've been very disheartened with the whole questionnaire because they've felt that their child wasn't performing but actually, you know if you took the questionnaire away as a Health Visitor I wouldn't have had concerns about that. (HP)

They noted that there is nothing on the questionnaire to reassure them that it is looking at a range of ability. Parents, too, suggested that there should be a caveat on the ASQ‐3 to say: “Don't worry if your child cannot do all of these ….”

### 3.7 Inconsistency in usage

At the time of the study, the ASQ‐3 was being used in the four study sites as a tool for health visitors and nursery nurses when assessing individual children's development at their 2‐year review. It was not then being used to collect data to inform a public health outcome measure.

Our findings from the focus groups showed a wide variation, both across and within sites, in how the ASQ‐3 was being used. This appeared to result from two major issues: One is how it has been introduced conceptually to HPs (at management level), and the other is associated with training. In Area 1, the approach was to regard it as the parents' tool and to be only one part of the child health and development assessment; it was not the assessment itself:

what we've tried to say to health visitors is that this is a parents' questionnaire, you know, it's a tool and that health visitors and nursery nurses must do their own assessment of the child first and then do a comparison with their findings with the parents. (HP)

However, in complete contrast, in another of the four research areas, a newly qualified member of staff reported that the ASQ‐3 was the main part: “this to me was sold to me ‘this is what you do now’ … this is going to be more the focus of newly qualified health visitors, yeah. … It's not like, we are not being told to do it in, like combining what we did, how we did it the old way” (HP).

Moreover, there is broad variation in usage between these two extremes, even within areas. The differing approaches mean it would be unlikely that all parents had a similar experience of the review and ASQ‐3 and that any recorded scores accurately reflect children's abilities in a standardized way.

For example, variations included it being used in the home, in clinics, with parents, put to one side, and used by health visitor or community nursery nurse. In terms of scoring, in some sites, HPs were specifically directed not to score in front of the family; in others, it was scored in front of parents, but only below‐threshold scores were discussed. In yet other settings, HPs scored the ASQ‐3 at the review with the parents and used the score to clarify and discuss any issues or concerns. Furthermore, there was evidence of misunderstanding of the scoring system of the ASQ‐3 by HPs; for example, one HP reported that she puts “not yet” if a child has not tried an activity rather than correctly adjusting the domain score. This could potentially lead to overreporting or underreporting of developmental delay. Referrals and reviews following assessment with the ASQ‐3 also differed by area. All of these variations might be accounted for by the substantial differences reported in HPs' ASQ‐3 training.

HPs also informed us of problems in reporting on the assessment and scores relating to time availability, access to a suitably adapted electronic child health information system, access to computers and internet, and overreliance on hard copy (personal child health record).

## 4 DISCUSSION

The ASQ‐3 has been shown internationally to be a valid parent‐completed measure (e.g., Kerstjens et al., 2009; Klamer, Lando, Pinborg, & Greisen, 2005; Kovanen, Maatta, & Heinonen, 2000; Richter & Janson, 2007). This study sought to explore parents' and HPs' experiences and perceptions of using the ASQ‐3 as part of the 2‐year review, prior to its introduction as a population measure in England. Other qualitative studies of parents' perspectives have also found the ASQ to be acceptable (Armijo et al., 2015; Singh et al., 2017); however, these tend to be adjunctive to validity studies and do not report in depth on parents or HPs' perspectives as we report here. In England, the aim of implementing the ASQ‐3 is to provide data that can be used at a population level for observation of child development with the potential to link to other administrative data such as school readiness measures, providing a child public health approach to development that can be universally adopted in England.

Overall, using the ASQ‐3 was largely unproblematic for parents, and they enjoyed using it with their children both as a stimulus for trying activities and for providing a sense of reassurance that their child is meeting milestones. However, the corollary to this was that it made some parents anxious about their child's development, especially when the child was unable to demonstrate specific abilities. This could lead parents to overvalue their child's developmental performance to avoid “failure.” HPs were aware of this too. However, as both parties welcomed the partnership approach provided by the ASQ‐3, there is provision in the system for anxieties to be managed and allayed. Indeed, research suggests that using a parent‐led developmental assessment tool can lead to more communication between a parent and practitioner about development and development concerns (e.g., Sices et al., 2008).

Some parents, including the parent engagement group, found the use of American English unfamiliar and awkward to use. As a result, we undertook some additional work to “translate” the ASQ‐3 (and the ASQ‐SE) into British English, and we took the opportunity to clarify the ASQ‐3 response options on the front cover and to address parents' anxiety by adding a “Do not worry if …” clause. Brookes, the ASQ‐3 publishers, approved the changes, and the revised measures have been implemented into the HCP across England. We have also provided feedback on concerns about specific questions to the ASQ‐3 creators to inform future development of the measure.

A key finding was that ASQ‐3 was used in different ways in the four sites and that training varied widely across England and led to inconsistent approaches to using the tool and scoring. Aside from overreferral or underreferral, this could distort the population picture of development at 2–2.5 years that could result in (or indeed explain) inconsistent and inequitable referral and service provision. A further development from the research has therefore been to provide two open‐access e‐learning modules for HPs, giving a consistent approach to the use and accurate scoring of the measures. Development of the e‐learning drew heavily on the findings of the study to address issues raised by both parents and professionals. This is available via e‐Learning for Healthcare website (http://www.e-lfh.org.uk/programmes/ages-and-stages-questionnaires/).

Recording of the ASQ‐3 is now part of the requirement for child health profiles across England, although the uptake is currently not readily available through the Child Health Profiles online. Recording children's scores and outcomes was a hurdle for HPs given that various electronic child health systems are in place in England (e.g., RIO, System1). With the development of the digital personal child health record (e‐Redbook) and its implementation, it would be advisable if all routine data such as the ASQ scores could be managed through a single system such as this.

### 4.1 Strengths and limitations of the study

Whilst this study was undertaken across England, the purposive sampling method cannot be representative of all parents or health care professionals, and therefore, this should be seen as a limitation. However, the strength of the study is the depth and breadth of qualitative data that have rarely been collected from parents in the United Kingdom on this topic, and the independent and systematic coding of the data. The findings have been widely accepted by the Department of Health and Social Care for England as well as the authors of the ASQ to encourage wider uptake of the 2‐year review.

In conclusion, this study of parents' and professionals' views on the acceptability and understanding of the ASQ‐3 has improved awareness of how parents and HPs can work in partnership to assess children and identify potential problems at the 2‐ to 2.5‐year stage of development. We have highlighted that parents are broadly in favour of the ASQ‐3 as it enables them to assess their own child and to work in partnership with HPs. It has also highlighted issues related to variation in use, training, and the language used in the ASQ‐3, which resulted in a rapid response by the Department of Health and Social Care for England and Health Education England to ensure that additional tools and guidance were put in place. The next steps will be to monitor ASQ‐3 uptake at 2–2.5 years and establish the evidence for variations in development among 2‐year‐olds in England and the impact this has on school entry and future outcomes.
